# Validation of Picture Free and Cued Selective Reminding Test for Illiteracy in Lima, Peru

**DOI:** 10.1177/15333175221094396

**Published:** 2022-04-24

**Authors:** Rosa Montesinos, Jose F. Parodi, Monica M. Diaz, Eder Herrera-Perez, Elizabeth Valeriano-Lorenzo, Ambar Soto, Carolina Delgado, Andrea Slachevsky, Nilton Custodio

**Affiliations:** 1 Unidad de diagnóstico de deterioro cognitivo y prevención de demencia, Instituto Peruano de Neurociencias, Lima, Perú; 2 Unidad de Investigación, Instituto Peruano de Neurociencias, Lima, Perú; 3 Centro de Investigación del Envejecimiento, Facultad de Medicina, 471934 Universidad de San Martín de Porres, Lima, Perú; 4 Department of Neurology, 4321 University of North Carolina at Chapel Hill, Chapel Hill, NC, USA; 5 Facultad de Salud Pública y Administración, 33216 Universidad Peruana Cayetano Heredia, Lima, Perú; 6 Grupo de Investigación Molident, 33225 Universidad San Ignacio de Loyola, Lima, Perú; 7 Faculty of Psychology, Autonomous University of Madrid, Madrid, Spain; 8 Departamento de Neurología y Neurocirugía, Hospital Clínico Universidad de Chile, Santiago, Chile; 9 Departamento de neurociencia, 37619 Facultad de medicina Universidad de Chile, Santiago, Chile; 10 Neuropsychology and Clinical Neuroscience Laboratory (LANNEC), Physiopathology Department, ICBM, Neurosciences and East Neuroscience Departments, 37619 University of Chile School of Medicine, Santiago, Chile; 11 Geroscience Center for Brain Health and Metabolism (GERO), 37619 University of Chile School of Medicine, Santiago, Chile; 12 Memory and Neuropsychiatric Clinic (CMYN), Neurology Department, 37619 Del Salvador Hospital and University of Chile School of Medicine, Santiago, Chile; 13 Neurology Unit, Department of Medicine, Alemana Clinic, Universidad del Desarrollo, Santiago, Chile; 14 Servicio de Neurología, Instituto Peruano de Neurociencias, Lima, Perú; 15 Escuela Profesional de Medicina Humana, 33222 Universidad Privada San Juan Bautista, Lima, Perú

**Keywords:** Alzheimer´s disease, dementia, educational level, illiteracy, free and cued selective reminding test, Latin America, Peru

## Abstract

Dementia in Latin America is a crucial public health problem. Identifying brief cognitive screening (BCS) tools for the primary care setting is crucial, particularly for illiterate individuals. We evaluated tool performance characteristics and validated the free and total recall sections of the Free and Cued Selective Reminding Test-Picture version (FCSRT-Picture) to discriminate between 63 patients with early Alzheimer’s disease dementia (ADD), 60 amnestic mild cognitive impairment (aMCI) and 64 cognitively healthy Peruvian individuals with illiteracy from an urban area. Clinical, functional, and cognitive assessments were performed. FCSRT-Picture performance was assessed using receiver operating characteristic curve analyses. The mean ± standard deviation scores were 7.7 ± 1.0 in ADD, 11.8 ± 1.6 in aMCI, and 29.5 ± 1.8 in controls. The FCSRT-Picture had better performance characteristics for distinguishing controls from aMCI compared with several other BCS tools, but similar characteristics between controls and early ADD. The FCSRT-Picture is a reliable BCS tool for illiteracy in Peru.

## Introduction

The number of people with dementia in Latin American (LA) is estimated to increase from 9.5 million in 2015 to 40 million in 2050.^1^ Dementia in LA is a crucial public health problem given longer life expectancies predicted throughout the region with persisting low socio-economic and educational levels.^2^ One review of 8 population-based cohort studies in LA, including Brazil, Cuba, Chile, Peru and Venezuela, found that dementia prevalence among elderly patients was nearly 7.1%, similar to rates published from high-income countries.^3,4^ However, the prevalence of dementia among individuals 65-69 years of age is significantly higher in LA countries compared to high-income countries (2.65% vs 1.0% in women; 2.27% vs 1.6% in men).^4^ In Cercado de Lima, a district in the capital city of Peru, one study reported a dementia prevalence of 6.85% among 1532 individuals 65 years or older with Alzheimer’s disease dementia (ADD) being the most common dementia sub-type (56.3%).^5^

Evidence suggests that 40-90% of people with dementia have not yet been formally diagnosed,^6^ which may be due to various diagnostic modalities needed to arrive at the diagnosis that are often lacking in regions of LA. Therefore, identifying the barriers to dementia diagnosis may help explain underdiagnosis rates. In LA, primary care physicians are insufficiently trained to diagnose dementia and have even less experience in diagnosing mild cognitive impairment (MCI), a pre-dementia state.^7-9^ Moreover, few countries in the LA region have executed a national dementia plan (only the governments of Argentina, Brazil, Chile, Costa Rica and Mexico have implemented an active national dementia plan to-date). Although these plans delineate the care and management of patients with dementia for each respective country, the plans also recommend that primary care physicians refer patients suspected of having dementia to specialists (ie geriatricians, a psycho-geriatricians, or neurologists) to confirm the diagnosis, creating further difficulties given shortages of specialists trained in diagnosing dementia in many of these regions.^1,10^

Cognitive impairment (further categorized as dementia, MCI or subjective cognitive complaints but with normal cognition on neuropsychological testing) is diagnosed by clinical history and administration of one neuropsychological tool or multiple brief cognitive screening (BCS) tools. The next step is to confirm the etiology of cognitive impairment (ie dementia secondary to neurodegenerative disease, vascular dementia or mixed dementia) by clinical examination, neuroimaging and a complete neuropsychological test battery if needed.^1,11^ A key barrier to determining the etiology of dementia in LA is the scarcity of neuropsychologists trained in administration and interpretation of these test batteries and time constraints in the primary care setting limiting application of these cognitive assessment tools.^2^ In addition, the majority of cognitive assessment tools were created for patients from high-income countries with few having been adapted to the cultural, linguistic, educational and literacy aspects of LA, likely overestimating the true prevalence of dementia in the region.^12^

Validating cognitive assessment tools with adequate psychometric properties is crucial for populations with various educational and literacy levels, particularly for illiterate individuals.^13^ In Peru, cognitive assessment tools (Clock Drawing Test (CDT)-Manos-Wu’s version,^14,15^ Addenbrooke’s Cognitive Examination (ACE),^16^ Memory Alteration Test (M@T),^17^ the Peru Coin Test,^18^ INECO Frontal Screening (IFS)^19^ and Rowland Universal Dementia Assessment Scale-Peru version (RUDAS-PE)^20(p)^) have only been validated for individuals with a mean educational level of 10 years living in the capital city, Lima. One study found that the M@T had adequate discriminatory ability to distinguish cognitively healthy individuals from amnestic MCI and ADD in early stages in an urban population with less than 4 years of education in Lima, Peru.^21^ Another study validated the RUDAS for distinguishing MCI and early-stage dementia in an illiterate urban population of Lima.^22^ However, currently validated tools for illiterate persons do not assess many of the cognitive domains affected in various dementia sub-types, limiting their ability to diagnose dementia sub-types. Moreover, there are no screening tools for episodic memory impairments validated for use in illiterate persons. Therefore, validating cognitive assessment tools adept at evaluating episodic memory and specific for illiterate populations of LA is needed given episodic memory impairments are one of the first presenting symptoms of aMCI and ADD.

The national illiteracy rate among Peruvians over 15 years of age is high (5.6%), with higher rates occurring in rural geographic areas of Peru (14.5*%* vs 3.4% in urban areas). An even greater illiteracy rate exists in adults over 60 years of age in rural areas (41.6% vs 12.3% in urban areas) and among females (25.0% vs 7.5% in males).^23^ Validated and standardized instruments for the assessment of cognition and functional status in populations with low educational levels and illiteracy are lacking, as are instruments for rural, indigenous and non-Spanish-speaking populations.^13,24^ The Free and Cued Selective Reminding Test (FCSRT) was created by Grober and Buschke,^25^ as an episodic memory test that specifically evaluates memory and has good discriminative ability to predict the development of ADD 5 years before its clinical onset among elderly subjects, with little influence of schooling factors making it an ideal test to detect ADD among illiterate individuals.^26^ The FCSRT has been validated for populations in Italy,^27,28^ Portugal,^29^ United States,^30^ Argentina^31^ and Chile,^32,33^ and is a tool that may allow timely assessment of episodic memory in illiterate patients with amnestic MCI and early ADD in the primary care setting.

Our study sought to evaluate the validity of the free and total recall sections of the FCSRT-Picture version in an urban illiterate population attending a primary care clinic in the community of Ventanilla, located in the Callao district of Lima, Peru. We determined the sensitivity, specificity, discriminative ability, positive and negative predictive values, and cut-off scores of the free and total recall sections of the FCSRT-Picture version compared with the Rowland Universal Dementia Assessment Scale-Peru version (RUDAS-PE) among illiterate individuals with normal cognitive status, amnestic MCI (aMCI) and ADD.

## Methods

### Study Design and Population

This was an observational, cross-sectional study that aimed to validate the psychometric properties of the FCSRT-Picture version among urban illiterate individuals in Callao, a region surrounded by Lima, the capital city of Peru. We sought to compare the performance of the free and total recall sections of the FCSRT-Picture version of illiterate cognitively healthy individuals and patients with amnestic MCI (aMCI) and early ADD.

The study was conducted in the health centers of the Regional Health Directorate of Callao (“DIRESA Callao”). Considering the socioeconomic distribution of residents and health facilities in the area, the district of Ventanilla was chosen, within the Pachacutec healthcare network, which spans several health centers including: “*Centro Base Perú-Corea-Pachacutec”, “Centro de Salud 03 de Febrero”, “Centro de Salud Santa Rosa de Pachacutec”, “Centro de Salud Bahia Blanca”,* and *“Puesto de Salud Ciudad Pachacutec”.*

Inclusion criteria for enrollment into this study included: male and female individuals over 50 years of age; illiterate individuals, defined as a person with less than 1 complete year of formal education and inability to read and write); individuals grouped into 1 of 3 cognitive categories: cognitively healthy, aMCI or early ADD described further below. We excluded those individuals who had difficulty completing cognitive testing due to hearing, visual or other physical impairments that could interfere with cognitive testing; those whose native language was that other than Spanish; those with functional literacy (defined as those with less than 4 years of formal education prior to age 15 and can read, write, perform mathematical calculations and are socially functional); those with a diagnosis of moderate to advanced dementia, concomitant cerebrovascular disease, developmental disabilities, traumatic brain injury, depression (screened using Beck Depression Inventory-II [BDI-II] results), history of addiction or substance abuse, previous diagnosis or symptoms of psychiatric illness (bipolar disorder, psychosis, schizophrenia and personality disorders).

### Ethical Considerations

The research protocol was approved by the Postgraduate Section of the Faculty of Human Medicine and the Ethics Committee of the San Martín de Porres University in Lima, Peru. Once the document was accepted, letters were sent to the appropriate authorities of DIRESA Callao in order to initiate the project in each respective health center.

### Study Procedures

After obtaining institutional permission, the nursing staff of the respective health centers were approached, informing them of the purpose and expected benefits of the study, and seeking their support for study implementation. Before initiating the study procedures, we conducted a training phase for study personnel. During the training phase, study interviewers (psychology students, primary care physicians and geriatric resident physicians) and neuropsychologists of the Diagnosis of Cognitive Impairment and Prevention of Dementia Unit (UDDCPD) of the Peruvian Institute of Neurosciences (IPN) were trained on study protocol methodology and appropriate administration of neuropsychological tests and other cognitive tools used in this study, the MMSE, CDT-Manos-Wu’s version, Pfeffer Activities of Daily Living Questionnaire (PFAQ), BDI-II, Modified Hachinsky Score, RUDAS-PE, and the FCSRT-Picture version. Study neuropsychologists applied standardized criteria for the diagnosis of aMCI and early ADD described below.

We completed an outreach campaign in the Ventanilla district. Potential participants were selected in 2 ways: a) via presentations on cognitive health (people who did not routinely receive care at the health centers where the study took place) and b) patients who regularly received care in one of the affiliated health centers. Individuals potentially eligible for the study were selected by simple random sampling. The study procedures were explained in detail, and all interested individuals signed (or marked their fingerprint to signify their approval of study participation) an informed consent form, previously reviewed and accepted by the appropriate regulatory authorities. The following study procedures were then applied during the study visit at one of the healthcare centers.

#### Clinical evaluation

An interviewer trained in the study protocol and procedures applied a demographic data questionnaire, completed a brief neurological examination, anthropometric and blood pressure measurements and recorded medical comorbidities and medications received the day before the evaluation.

#### Gold Standard for diagnosis of aMCI or early ADD

**Figure 1.**
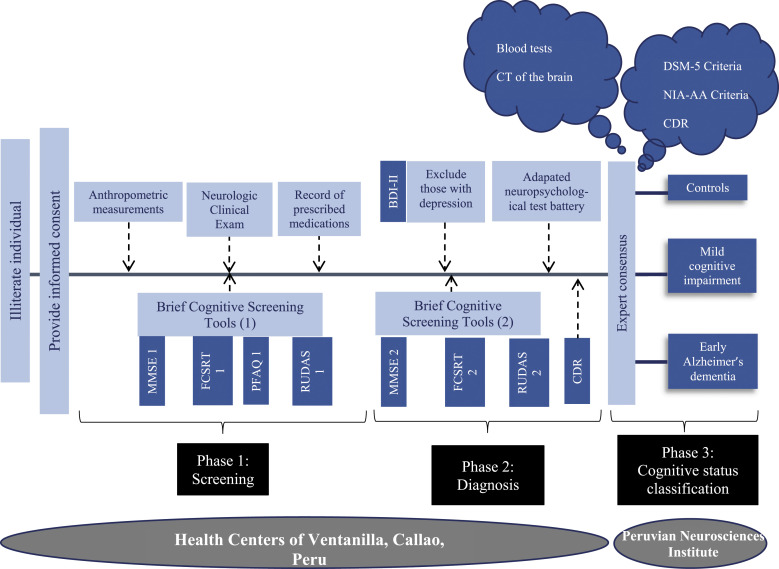
Flowchart of steps for diagnosis of Mild Cognitive Impairment or early Alzheimer’s dementia. Abbreviations: BDI-II: Beck Depression Inventory-II; CDR: Clinical Dementia Rating scale; CT: Computerized Tomography; DSM-5: Diagnostic and Statistical Manual of Mental Disorders-5; FCSRT: Free and Cued Selective Reminding Test (picture version); MMSE: Mini Mental State Exam; NIA-AA: National Institute on Aging-Alzheimer’s Association; PFAQ: Pfeffer Activities of Daily Living Questionnaire; RUDAS: Rowland Universal Dementia Assessment Scale (Peruvian version).

Individuals enrolled in the study underwent the following successive assessments in 3 phases: screening, dementia diagnosis and diagnostic classification phases (Figure 1).

Stage 1 (Screening Phase):

The screening phase was used to screen for presence of cognitive impairment. In the first phase (screening phase), individuals underwent the following tests: FCSRT-Picture, MMSE, RUDAS-PE, CDT-Manos-Wu’s version and PFAQ. Individuals who performed below the MMSE and PFAQ score-cutoffs for cognitive impairment established for this research protocol (MMSE <18 for illiterate individuals^34^; and PFAQ >7^35^), then entered into the second stage (dementia diagnosis phase) in order to determine cognitive impairment severity.

Stage 2 (dementia diagnosis phase):

Those that entered the second stage underwent an evaluation by a second evaluator (neurologist or geriatrician of the UDDCPD-IPN) different than the first evaluator. When the MMSE demonstrated cognitive impairment (score < 18) in the first phase, both the MMSE and FCSRT-Picture was repeated by a different examiner in the second phase (neurologist or geriatrician from the UDDCPD-IPN). Those who scored < 18 on the MMSE in the second phase were considered to be cognitively impaired. The same versions of the MMSE and FCSRT-Picture were administered in the first and second phases. The BDI-II, Modified Hachinsky index, were also administered in this phase.

In this second phase, the CDR, DSM-5 and NIA-AA criteria, was based on a neuropsychological battery of tests adapted for low education and illiterate individuals. Impairment in each cognitive domain was defined as a cognitive domain score 2 standard deviations (SD) below the mean. The CDR was performed by 2 evaluators simultaneously (neuro-rehabilitation specialist and a neuropsychologist) from the UDDCPD-IPN, who each scored the patient in the same interview blinded to one another’s’ score. In this second phase, the study groups were classified using the criteria for dementia and MCI using: the DSM-V dementia criteria,^36^ the NIA-AA criteria for MCI criteria.^37^ Dementia severity was established using the Clinical Dementia Rating (CDR) criteria^38^ in the AD group. In this study, only cases with CDR = 0 (control group); CDR = .5 (amnestic MCI group), CDR = 1 (early-stage Alzheimer’s disease dementia group) and CDR = 2 (moderate stage Alzheimer’s disease dementia group) were considered. The CDR was applied to both study subjects and their caregivers/companions. Cases in which there was doubt as to the diagnosis were resolved by consensus between neurologists, geriatricians, neuro-rehabilitation specialists and neuropsychologists of the UDDCPD-IPN. Importantly, the FCSRT-Picture administered as part of this study were not used to classify patients into these groups and scores were not revealed to the raters who classified participants into each group.

Stage 3 (diagnostic classification phase):

In the third stage (diagnostic classification phase), we included blood test results (blood count, glucose, creatinine, transaminases, vitamin B12, thyroid hormones, RPR and HIV-ELISA) and non-contrast brain CT scan, to exclude cases of vitamin B12 deficiency, hypothyroidism, vascular dementia, etc., to arrive at the final cognitive status categories:

1. Control group: individuals without cognitive impairment (MMSE score greater than 18)
2. Amnestic MCI (aMCI) group: Individuals with clinical and neuropsychological criteria of aMCI using NIA-AA criteria.^37^
3. Early ADD group: Individuals with clinical and neuropsychological criteria compatible with early ADD using NIA-AA criteria.^39^

#### Cognitive Tests administered

##### Free and total recall sections of the picture version of the FCSRT.

The FCSRT was created by researchers Ellen Grober and Herman Buschke^25^ based on the idea that when a subject performs episodic memory tasks, he or she will benefit from cues that help with memory encoding.^26^ In this study, we apply a version of FCSRT-Picture version is a selective test of episodic memory (verbal memory) adapted to LA populations.^33^ The task consists of 6 sequential phases: (1) image identification, (2) interference tasks, (3) free recall, (4) cued recall of images that were not previously recalled, (5) selective reminding of images that were not previously recalled with a category cue, and (6) delayed recall at approximately half an hour (both free and cued recall). Phases 2 through 5 are repeated 3 times during the learning process.

The phases are as follows:

(1) Identification: a sheet with 4 images is placed in front of the study subject and he/she is asked to name them aloud according to the category to which the images belong. The sheet is then removed and the subject is asked to recall the figures that were shown on the sheet. If the subject does not recall any of the figures on the sheet, the sheet is shown again and the subject is asked to repeat and point out the figure that he/she could not remember. This procedure is repeated with the 3 additional sheets. If the subject is unable to identify any of the 4 images, the test is stopped.
(2) Interference: The subject completes a serial subtraction task for 20 seconds (for example, counting down from 40 to 1) to prevent subvocal repetition.
(3) Free recall: The subject names the images he/she remembers from the first phase, in any order within 90 seconds. The task is stopped if the subject does not respond for 15 seconds. Before the second and third free recall trials, we also performed the interference task described previously.
(4) Cued recall: After each free recall trial, we proceed to the cued recall task, only for those items that have not been spontaneously recalled. For each image not recalled, a category cue is given.
(5) Selective reminding: In cases where the image was not recalled with the cues, the participant is reminded of the image with a category cue. Selective reminding was only performed in the first 2 trials. Since this protocol intended to evaluate a brief cognitive screening test (less than 15 minutes), we did not evaluate delayed recall at 30 minutes due to the added length of administration time of the test.

##### Mini Mental State Examination (MMSE)

The Mini Mental State Examination (MMSE)^34^ is a test that briefly assesses orientation (in time and space), immediate recall (or 3-word recall), attention and calculation, delayed recall, language (naming, repetition, reading, following commands and writing) and constructive praxis. The cutoff score on the MMSE for suspected dementia was adjusted for years of education: 27 for individuals with more than 7 years of education, 23 for those with 4 to 7 years of education, 21 for those with 1 to 3 years of education, and 18 for illiterate individuals.^5^ Since all subjects in this study had illiteracy, an MMSE cut-off of 18 was utilized for dementia.

##### Rowland Universal Dementia Assessment Scale – Peru version (RUDAS-PE)

The RUDAS-PE^22^ includes assessments of 6 cognitive domains, starting with memory registration, followed by visuospatial orientation, motor praxis, visuospatial construct, judgment, short-term recall and language. In the memory registration phase, the individual is asked to remember a list of things to buy in a store: coffee, oil, eggs and soap; ensuring that it is registered by repeating the list up to a maximum of 5 times. Next, to assess visuospatial orientation, the individual is asked to show or point to different parts of his own and the evaluator’s body. To assess motor praxis, the individual is asked to perform alternating and successive hand postures, consisting of making a fist with one hand and an open hand with the other. Then, the participant copies a cube printed on a sheet of paper. To evaluate judgement, a hypothetical situation is given to the participant and they must find possible solutions to a situation in which the person is asked how they would cross a street safely if there were no traffic light or pedestrian crossing. To evaluate short-term memory recall, the individual is asked to name the list of things to buy in the store from earlier. Finally, to evaluate language, the individual is asked to say as many different names of animals as possible in 1 minute.^22^

The Clock Drawing Test (CDT)-Manos-Wu’s version, Pfeffer Functional Activities Questionnaire (PFAQ), Beck Depression Inventory, second version (BDI-II) and Modified Hachinski index were administered to all participants.

### Data Processing and Statistical Analyses

All data collected was organized into a database. Statistical analyses were performed by a statistician who was blinded to the results of the gold standard cognitive tests.

#### Demographic Characteristics and Cognitive Performances

General and cognitive characteristics of the 3 diagnostic groups (controls, aMCI and early ADD groups) were analyzed using descriptive statistics for categorical (frequency and proportion) and numerical (mean and SD) variables. We performed pairwise comparisons using Chi-squared test for the categorical variable (sex), and we used ANOVA and Bonferroni tests to perform multiple comparisons between diagnostic groups for continuous variables (age and brief cognitive tests scores).

#### Content Validity

To assess content validity, 5 dementia experts (specialists with at least 2 years of experience in cognitive and neuropsychological assessments) completed an ad hoc questionnaire to assess the content validity of the FCSRT-Picture version. The experts were asked about the test’s ability to assess episodic memory, its ability to measure the corresponding indicator and administration and scoring instruction clarity. Any disagreements were discussed by the research team (neurologists, geriatricians, psychiatrists, neuropsychologists and rehabilitation physician) and any changes to the proposed version of the test were defined by consensus (Supplementary Material I). Based on this expert evaluation, the Chilean version^33^s was deemed acceptable for a Peruvian population because the images could be understood by individuals of the community where this study took place and adequately measure episodic memory without significant influence from educational level.

#### Reliability

To evaluate test reliability, we calculated Cronbach’s alpha coefficient using the FCSRT-Picture version results obtained in the first phase (dementia screening phase) as a measure of the test’s homogeneity and internal consistency. To evaluate the test-retest reliability, we estimated Lin’s concordance and correlation coefficients comparing the FCSRT-Picture version measured in the first and second study phases (screening vs dementia diagnostic phases).

#### Convergent Validity

Convergent validity was assessed using correlations between FCSRT-Picture scores and other cognitive assessment tool scores (RUDAS-PE) and functional assessment scores (PFAQ and CDR) among individuals evaluated in the second phase (those who underwent the “gold standard” neuropsychological battery). Spearman correlation coefficients were applied because of the non-normal distribution of the data.

#### Criterion Validity

Criterion validity was assessed using receiver operating characteristic (ROC) analyses. We calculated the area under the curve (AUC) of the FCSRT-Picture scores to discriminate diagnostic subtypes (control, aMCI and early ADD groups) by pairs. The optimal cut-off points for the FCSRT-Picture were determined using data from participants who completed the first 2 study phases to calculate the diagnostic accuracy of the test. Together with the ROC analysis, the Youden’s Index was used to derive the optimal cutoff values for each test.

## Results

**Figure 2.**
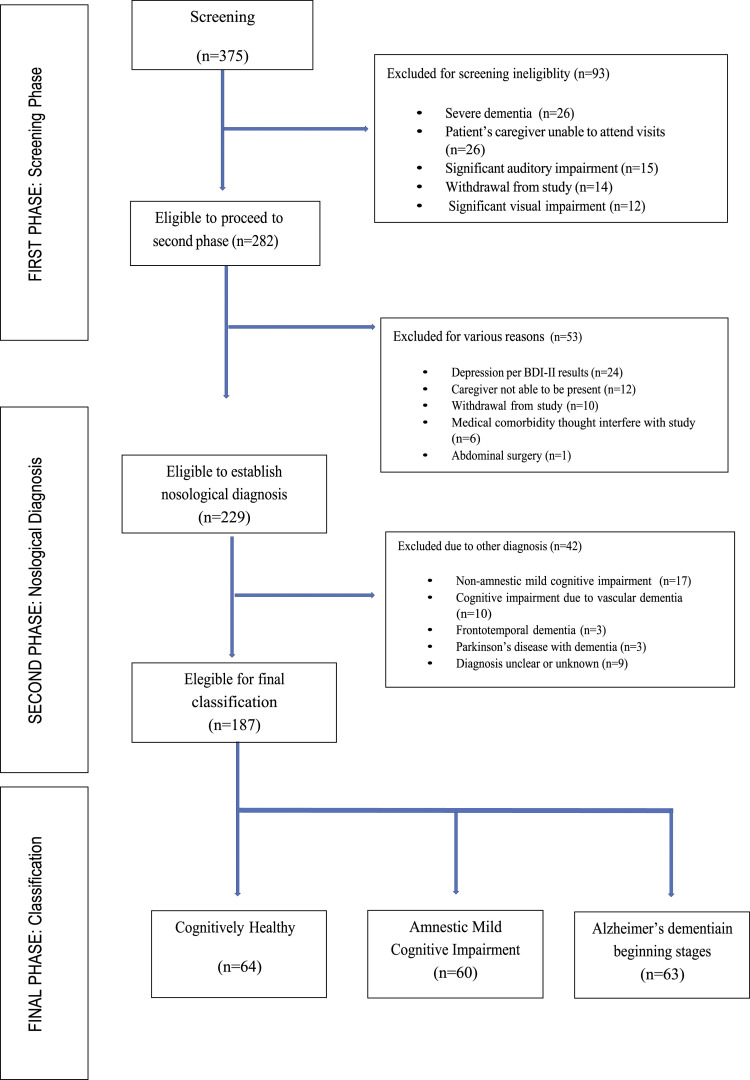
Flow chart of participants from Ventanilla Health Centers, Callao, Lima. 2018-2019.

During the screening phase, 375 individuals over 50 years of age were evaluated, and 93 were excluded for various reasons. In total, 282 entered the second phase (diagnostic phase). In this second phase, 53 individuals were excluded for other reasons, with a total of 229 individuals over 50 years of age enrolled into the study. Forty-two of these individuals were excluded from the study because they were classified as non-amnestic MCI (n = 17), vascular dementia (n = 10) and other types of dementia (15 patients, including cases of frontotemporal dementia [FTD], dementia with Parkinson’s disease and unknown etiology of dementia). The final sample analyzed included 187 subjects (64 cognitive healthy, 60 aMCI, 63 early ADD; Figure 2).

### Study Population Characteristics

We included 105 women (56.2%) with a similar proportion between the 3 groups (55.6% in the early ADD group, 56.7% aMCI and 56.3% controls) and no significant difference between-groups. The mean±SD age of the overall sample studied was 70.2 ± 3.8 years; the aMCI group was significantly younger compared to the other 2 groups (P < .05). The aMCI group (P = .000) and the cognitively healthy group (P = .000) were both younger than the ADD group. The aMCI group was younger than the cognitively healthy group, but this difference did not reach statistical significance (P = .794).

The ADD group performed significantly worse on all cognitive screening tools administered, including the MMSE, RUDAS-PE, and the free and total recall sections of the FCSRT-Picture version (P = .000 for each test), compared to the 2 other groups (aMCI and controls). The mean MMSE score of the early ADD group was 10.1 ± 1.6, 17.9 ± 1.6 in aMCI group and 20.2 ± 1.5 in the control group. In the RUDAS-PE, patients with early ADD had a mean score of 15.0 ± 2.2, aMCI 20.4 ± 1.4 and controls 23.9 ± .9. The mean scores of the free recall section of the FCSRT-Picture version in the early ADD group was 7.7 ± 1.0, aMCI was 11.8 ± 1.6 and cognitively healthy was 29.5 ± 1.8; while the mean total recall score of the FCSRT-Picture version of the early ADD group 17. ± 1.4, 21.0 ± 1.8 in aMCI group and 44.1 ± 2.2 among the cognitively healthy group. In all cases, the mean cognitive screening tool scores were significantly different between the 3 study groups (Table 1).

**Table 1.** ROC areas percentages and confidence intervals by brief cognitive screening tool.

BCS	Control vs aMCI	aMCI vs AD
RUDAS-PE	97.97 96.33 – 99.61	98.28 96.45 – 100
Free recall FCSRT-Picture	100 100 – 100	99.15 98.29 – 100
Total recall FCSRT-Picture	100 100 – 100	93.45 89.71 – 97.19

Abbreviations: BCS, brief cognitive screening; aMCI, amnestic mild cognitive impairment; AD, early Alzheimer’s dementia; SD, standard deviation; RUDAS-PE, Rowland Universal Dementia Assessment Scale, Peruvian version; FCSRT, Free and Cued Selective Reminding. The values for comparison between control and AD were omitted because all results were 100%.

### Reliability

Internal consistency was calculated based on results from the 187 participants who completed the third phase of the study. The Cronbach’s alpha coefficient for free recall of the FCSRT-Picture in illiterate older adults studied was .81; while the Cronbach’s alpha coefficient for total recall of the FCSRT-Picture in this population was .77. When one of the FCSRT-Picture figures was removed, the overall Cronbach’s alpha coefficient for free and total recall of FCSRT-Picture did not increase and decreased instead. All figures were had adequate internal consistency for the FCSRT-Picture version. The test-retest reliability of the free recall section of the FCSRT-Picture measured by intraclass correlation (ICC) was .959 and the correlation between first and second assessments was 92.6%. Domain-to-total correlations were above 49%. Similarly, the test-retest reliability of the total recall version of the FCSRT-Picture by ICC was .967 and the correlation between the first and second assessment was 93.4%. Domain-to-total correlations were all above 46% for the total recall section.

### Convergent Validity

Convergent validity was determined using Spearman’s correlation coefficient between free and total recall of the FCSRT-Picture version compared with the RUDAS-PE, PFAQ and CDR. The correlation coefficient of the free recall scores of the FCSRT-Picture version vs RUDAS-PE was .85 (SD 0.24; 95% CI). In addition, the correlation between free recall of the FCSRT-Picture version compared with the PFAQ was .81 (SD 0.34; 95% CI) and between the free recall of FCSRT-Picture version compared with the CDR was .92 (SD 0.36; 95% CI). We found no correlation between either of these FCSRT tests and age or sex. The correlation coefficient for total recall of FCSRT-Picture and RUDAS-PE was .89 (SD0.31; 95% CI); vs PFAQ: .88 (SD 0.11; 95%CI); and vs CDR: .91 (SD 0.19; 95%CI).

### Criterion Validity

**Figure 3.**
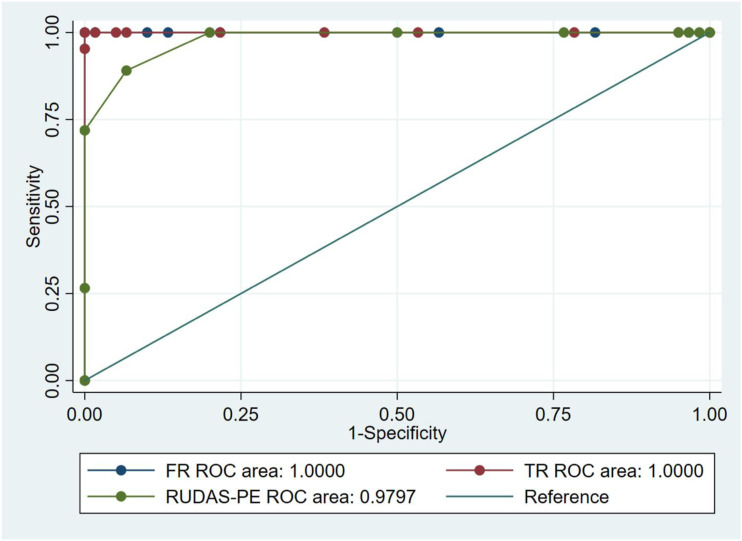
ROC curves of the free and total recall sections of FCSRT-Picture compared with the RUDAS-PE for controls vs amnestic Mild Cognitive Impairment.

**Figure 4.**
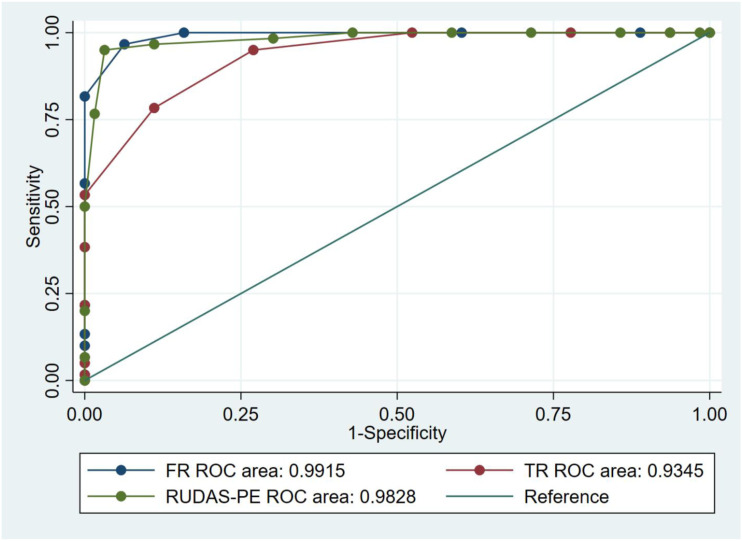
ROC curve of the free and total recall sections of FCSRT-Picture version compared with the RUDAS-PE for amnestic Mild Cognitive Impairment vs early Alzheimer’s disease dementia.

For each cognitive tool applied (RUDAS-PE and FCSRT-Picture version), the AUCs for ROC curves and the 95% confidence intervals were calculated between the 3 study groups, ie control vs aMCI group (n = 124), control vs early ADD group (n = 127) and aMCI vs early ADD (n = 123) (Table 1). AUC analyses comparing the controls and early ADD group showed that the RUDAS-PE and free recall and total recall of the FCSRT-Picture each reached a value of 1. Similarly, AUC analyses comparing controls and the aMCI group showed that the free recall and total recall of the FCSRT-Picture (AUC of 1 for each) were significantly better at discriminating the 2 groups compared with the RUDAS-PE (AUC .9797) (Figure 3). There were no significant differences between any of the assessed BCS tools for discriminating between aMCI and early ADD (Figure 4).

### Cut-Off Scores for Free and Total Recall Sections of the FCSRT-Picture Version for Illiterate Individuals in Peru

For the free recall section of the FCSRT-Picture, we found that the most adequate cut-off score for differentiating between aMCI and controls was a score of 16, early ADD vs controls was 10 and early ADD vs aMCI was also 10. For the total recall section of the FCSRT-Picture version, the optimal cut-off score for differentiating between aMCI and controls was 26, between early ADD and controls was 20, and between early ADD and aMCI was 19. For all cases, when we used the Youden index, the optimal cut-off point achieved ideal sensitivity, specificity and area under the curve (Youden index = 1.00 for both FCSRT total and free recall sections.

## Discussion

Our study found that the free and total recall sections of the FCSRT-Picture had better performance characteristics for distinguishing controls from aMCI when compared with the RUDAS-PE, and performed similar to the RUDAS-PE when comparing controls and early ADD. The AUCs of the free and total recall sections of the FCSRT-Picture were each higher than the RUDAS-PE for both early ADD vs controls and for aMCI vs controls. Internal consistency for both the free and total recall sections of the FCSRT-Picture was high and all figures contributed positively to the FCSRT-Picture. We found that the free and total recall sections of the FCSRT-Picture version is reliable and a useful episodic memory test in illiterate Peruvian individuals from an urban area.

This study is the first to validate the FCSRT-Picture version in illiterate individuals from an urban community. To date, only 2 studies have validated the FCSRT in a population with a low educational level and with a small proportion of illiterate individuals (one study in a university hospital in Coimbra, Portugal [mean educational level 4 years]^29^; and the other in a memory unit of 2 university hospitals in Spain with nearly 42% of the participants with less than a primary school level of education^40^). In both studies, the AUCs of the free recall section of the FCSRT-Picture to differentiate controls from aMCI approached a value of 1 (.818 in the Portuguese study and .990 in the Spanish study).^41^ Furthermore, other studies in middle-to highly-educated populations in Spain,^42^ Italy^28^ and Chile^32^ have demonstrated the same excellent discriminative validity of the “Word” and “Picture” versions of the FCSRT for distinguishing between controls and aMCI. Only in one study from Madrid, Spain was the AUC much lower on the free recall of the FCSRT-Picture version (.648) in a sample of patients with mean educational level of 12 years.^43^ Thus, the discriminative ability of the free recall FCSRT-Picture version to differentiate controls from early ADD and early ADD from aMCI is superior to the Peruvian version of the RUDAS-PE. The FCSRT-Picture version has several qualities that make it an effective cognitive assessment tool for evaluation of episodic memory, including its ease of administration by any health professional without needing specialized tools or training, well-tolerated and accepted by patients, easy to score, and can be applied in individuals with a low educational levels, although it is unknown if a language other than Spanish may independently influence test results. Moreover, given it uses verbal and visual cues which may enhance episodic memory,^44^ it may be a more adequate tool compared the RUDAS-PE in illiterate individuals.

In Peru, several cognitive assessment tools have been shown to have adequate psychometric properties for early stages of dementia and MCI^45^; but most of them have been adapted and validated for use in individuals with high educational levels.^12,46,47^ In low- and middle-income countries, manual labor is a common occupation of elderly populations from rural or urban areas of low socioeconomic strata, therefore, cognitive assessment tools that require reading and writing skills lose sensitivity and specificity in this population, leading to a high proportion of false-negative cases.^12,45-47^ In LA, the FCSRT (both “Word” and “Picture” versions) has been shown to be a reliable instrument with high accuracy for discriminating between early ADD, aMCI and cognitively healthy subjects, when applied to a population with a mean educational level of 12 years attending a specialized memory center in Chile.^32,33^ This emphasizes the importance of adaptation and validation of episodic memory assessment tools for this vulnerable population. Given the FCSRT-Picture version does not require the patient be able to read or use additional instruments such as pencil and paper, it can be easily administered to illiterate individuals with other cognitive tools, such as the RUDAS-PE or MMSE.

Internal consistency of the free recall section of the FCSRT-Picture is a psychometric property that has not been frequently evaluated in previously published studies, even in the original studies by the creators of the test.^48^ Cronbach’s alpha coefficient between .70 and .90 are considered ideal; but a value of .60 is considered acceptable. In our study, the internal consistency of the FCSRT-Picture was .81, while in an urban population with low educational levels (mean educational level of 4 years) from Coimbra, Portugal, the internal consistency of the free recall section of the FCSRT-Picture was .91^29^ and .84 in the “word” version evaluated in Milan, Italy in a population with a mean educational level of 8 years.^28^ In another study, the internal consistency was .82 for the FSCRT-Picture in a Chilean population with a mean educational level of 12 years.^32^ Therefore, our study has demonstrated an internal consistency similar to that of other published studies of low educational levels.

Criterion validity of the free recall section of the FCSRT-Picture version using Spearman’s correlations showed acceptable agreement with the RUDAS-PE (.85; SD 0.24; 95% CI) suggesting that the free recall of the FCSRT-Picture is a good cognitive predictor. Moreover, free recall of the FCSRT-Picture showed excellent predictive properties of functionality, based on the correlation between the free recall section of the FCSRT-Picture with the PFAQ (.81; SD 0.34; 95% CI) and with the CDR (.92; SD 0,36; 95% CI), similar to findings in a Chilean study comparing the psychometric properties of the “Word” and “Picture” versions of the FCSRT with several cognitive tests (MMSE, Addenbrooke’s Cognitive Examination-Revised and the CDR).^32^ Our findings were also similar to those of an Italian study, in which the “word” version of the FCSRT showed excellent correlations with conventional memory tasks (recall of a story and the Rey auditory-verbal learning test in its immediate and delayed recall version).^28^ Scores on the “Picture” version of the FCSRT are expected to be higher than the “Word” version because in the “Picture” version, both a visual and verbal code are encoded together, enhancing memory for that specific item. Therefore, it would be expected that the “Picture” version may perform better than the “Word” version if the person being tested were literate.^44^ The strong correlations with cognitive abilities and functionality demonstrate good criterion or convergent validity and indicate that the free recall section of the FCSRT-Picture version may be a good test for dementia assessment in illterate individuals.

However, there were no correlations between free recall of FCSRT-Picture and age and sex in our study, similar to findings from the Chilean study.^32^ This lack of correlation, however, was different from those reported by Grau-Guinea et al^49^ in a Spanish study with a mean educational level of 12 years using the “word” version of the FCSRT, where a significant negative correlation with age and positive correlation with years of education were observed. A similar finding was seen in a study in the United Kingdom that found a significant age effect when comparing the free recall section of the FCSRT-Word version with the visual association memory tasks.^40^ However, our study did not use the “word” version of the FCSRT and instead used the “Picture” version, given we studied illiterate individuals, therefore we are unable to adequately compare with previously published studies. We can propose that the scores obtained on the FCSRT-Picture version can be interpreted without needing to adjust for age and sex in the urban illiterate population of Lima, Peru.

Discriminative validity analysis of the cognitive screening tools studied revealed that all of the tests assessed were able to discriminate adequately between controls and ADD patients (AUC = 1 for free recall FCSRT-Picture, total recall FSCRT-Picture, RUDAS-PE). They discriminated between controls and aMCI patients (RUDAS-PE), but the free and total recall sections of the FCSRT may not be as sensitive for discrimination between early ADD and aMCI. This demonstrates that the free and total recall of the FCSRT-Picture versions are adequate tools that can be used in illiterate populations such as the one in this study on its own or as an adjunct to cognitive assessment tools such as MMSE or RUDAS-PE.

Although the educational level in the Peruvian population has improved substantially in recent years,^50^ nearly 6% of the population over 15 years of age still cannot read and write.^23^ Illiteracy significantly affects people living in rural areas (14.9% vs 3.5% in urban areas), predominantly in the Peruvian highlands (10.3% compared to 7.5% in the jungle and 3.1% on the coast), among women (8.7% vs 3.0% among men) and among people over 60 years of age (18.4% compared to .6% in the 15-19 age group). In addition, illiteracy more frequently affects those whose native language is that other than Spanish (ie Quechua, Aymara or Amazonian languages), who account for 16.1%, compared to 3.4% native Spanish-speakers with illiteracy.^23^ On the other hand, the prevalence of dementia is more than double in individuals with no/low education compared to those who can read and write, therefore, it has been proposed that low literacy acquisition is directly related to the probability of diagnosing dementia.^4,51^ In a study in Cercado de Lima, a district of Lima, Peru, 15.2% of cases of dementia occurred in illiterate individuals, while only 3.7% in individuals with at least 8 years of education.^5^ Similarly, an analysis of community-based studies conducted in LA observed a high prevalence of dementia among illiterate individuals (15.7%) compared with 7.2% among those who could read and write^4^; moreover, in a study conducted in Butantã, a community in the western part of Sao Paulo in Brazil, dementia was more prevalent among illiterate, unemployed and low-income individuals, accounting for 22.0%, 38.5% and 38.5% of dementia cases, respectively.^52^ One likely contributor to this high rate of dementia detected in illiterate persons is that they may perform worse on psychometric or cognitive tests used for identifying cognitive impairment associated with dementia,^53,54^ increasing the likelihood of diagnostic bias or confusion, leading to over-diagnosis of dementia.

A timely diagnosis of dementia requires strict and specialized protocols incorporating a series of assessments including clinical evaluations, a neuropsychological battery of tests, neuroimaging, bloodwork and cerebrospinal fluid (CSF) analysis.^55^ These evaluations can take more than 1 month to complete and incur high socioeconomic costs. Despite an increase in the life expectancy among Peruvians likely leading to a higher incidence of dementia, there are insufficient experts and specialized diagnostic centers to meet the needs of people that require cognitive evaluations. Additionally, the gold standard for diagnosis of ADD dementia requires the use of CSF biomarkers and neuro-imaging, which are invasive, expensive tests with limited practical utility as a diagnostic tool in primary care services.^56^ Primary care physicians are also not trained to effectively diagnose dementia in early stages as 2 prior studies have shown,^7,8^ highlighting the need to develop screening and diagnostic tools that are fundamentally brief and easy to administer in the primary care setting.

One challenge to diagnosing dementia is the clinical stage of MCI, usually occurring 1 to 5 years before reaching the dementia stage.^57^ MCI is characterized by high scores on subjective memory complaint questionnaires with no to little impairment of functional abilities.^57^ Episodic memory is the first and most severe cognitive domain affected in early ADD and aMCI, however non-amnestic MCI could also be a risk factor or prodrome of other types of dementia, such as vascular dementia, FTD and dementia with Parkinson’s Disease, among others. There is an unmet demand for additional non-invasive and/or cost-effective tests that can be utilized in primary care settings to identify individuals in preclinical (MCI) and early stages of dementia.^1,2^ However, we have demonstrated that the free and total recall sections of the FCSRT-Picture is able to better differentiate controls from aMCI compared with the RUDAS-PE, demonstrating its utility on its own in illiterate populations in Peru for this purpose.

Limitations of this research include the following. First, the FCSRT-Picture version should not be used in isolation to define ADD dementia nor other types of dementia. The FCSRT-Picture test itself only evaluates episodic memory and does not assess other cognitive domains, such as orientation, language, visuospatial skills and executive function, therefore, it may be unable to detect other forms of cognitive impairment, such as non-amnestic MCI, atypical ADD (frontal variant or posterior cortical atrophy) and other non-Alzheimer’s type dementia (ie FTD, vascular dementia, dementia with Lewy bodies and dementia with Parkinson’s disease). Third, our study was conducted in an adult population that regularly attends primary health care centers, increasing the likelihood that participants had a concern cognitive status concern, limiting extrapolation of results to a community-dwelling population. However, we ensured that the classification of cognitive status and neuropsychological assessments were performed by a different investigator than the investigator who administered the tests in the initial screening phase. Because our results for cognitive impairment screening were based on the second MMSE evaluation (second phase) and the tests did not vary between first and second test administration, we cannot eliminate the possibility of learning effect. Fourth, these results can only be extrapolated to illiterate individuals living in urban areas and whose native language is Spanish. The performance of the FCSRT-Picture in rural populations and among individuals with a native language other than Spanish, such as Quechua and Aymara, is unknown. Finally, this study does not include the evaluation of delayed recall 30 minutes after the initial evaluation, which could improve the diagnostic ability of the test; however, this would increase administration time, making it less feasible for use in the primary care setting.

## Conclusions

We found that the free and total recall sections of the FCSRT-Picture version is a valid neuropsychological test in an urban illiterate population of Lima with adequate psychometric properties for distinction between early ADD and cognitively healthy people and aMCI and cognitively healthy people. The test has better performance characteristics for distinguishing between controls vs aMCI when compared with the RUDAS-PE. The free and total recall sections of the FCSRT-Picture have a high discriminative capacity to differentiate cognitively healthy individuals from early ADD and from aMCI, respectively. Because the FCSRT-Picture is a cognitive screening tool that is easy to administer, fast and efficient, it is an adequate neuropsychological test for cognitive impairment that can be used in primary care centers by any health professional. It can be used as an adjunct to cognitive assessment tools, such as the MMSE or RUDAS-PE, or with a functional assessment tool, such as the PFAQ. By combining the FCSRT-Picture with a functional assessment tool, the diagnosis of aMCI and early ADD can be made in a population similar to ours. The tool is useful for distinguishing between healthy controls and aMCI while maintaining the ability to differentiate between aMCI and early ADD. Additional research is needed to evaluate the performance of the free and total recall of the FCSRT-Picture version in low-educated and illiterate individuals in rural communities, as well as its performance in low-educated and illiterate individuals with native languages other than Spanish.

## Supplemental Material

sj-pdf-1-aja-10.1177_15333175221094396 – Supplemental Material for Validation of Picture Free and Cued Selective Reminding Test for Illiteracy in Lima, Peru Click here for additional data file. Supplemental Material, sj-pdf-1-aja-10.1177_15333175221094396 for Validation of Picture Free and Cued Selective Reminding Test for Illiteracy in Lima, Peru by Rosa Montesinos, Jose F. Parodi, Monica M. Diaz, Eder Herrera-Perez, Elizabeth Valeriano-Lorenzo, Ambar Soto, Carolina Delgado, Andrea Slachevsky and Nilton Custodio in American Journal of Alzheimer's Disease & Other Dementias®

## Appendix

### Notation

ACE: Addenbrooke’s Cognitive Examination
ADD: Alzheimer’s disease dementia
aMCI: amnestic MCI
ANOVA: Analysis of Variance
BCS: brief cognitive screening
BDI-II: Beck Depression Inventory-II
CDR: Clinical Dementia Rating
CDT: Clock Drawing Test
CSF: cerebrospinal fluid
DIRESA: Regional Health Directorate
FCSRT: Free and Cued Selective Reminding Test
ICC: intraclass correlation
IFS: INECO Frontal Screening
IPN: Peruvian Institute of Neurosciences
LA: Latin America
M@T: Memory Alteration Test
MANOVA: Multivariate analysis of variance
MCI: mild cognitive impairment
MFE: Memory Failures of Everyday Life
MMSE: Mini Mental State Exam
NIA-AA: National Institute on Aging-Alzheimer’s Association
PFAQ: Pfeffer Activities of Daily Living Questionnaire
RUDAS-PE: Rowland Universal Dementia Assessment Scale-Peru version
SD: standard deviation
UDDCPD: Diagnosis of Cognitive Impairment and Prevention of Dementia Unit
