# Supplementary material for: Validation of Picture Free and Cued Selective Reminding Test for Illiteracy in Lima, Peru
Source: Am J Alzheimers Dis Other Demen. 2022 Apr 24;37:15333175221094396. doi: 10.1177/15333175221094396 (PMC10581140; doi:10.1177/15333175221094396)
Supplement: sj-pdf-1-aja-10.1177_15333175221094396 – Supplemental Material for Validation of Picture Free and Cued Selective Reminding Test for Illiteracy in Lima, Peru [file sj-pdf-1-aja-10.1177_15333175221094396.pdf]

**Supplementary Material II:**
**Description of other tests administered**

### Clock Drawing Test (CDT)-Manos-Wu's version

The CDT-Manos-Wu's version assesses the following cognitive skills: auditory comprehension, planning, visual memory, reconstruction of a graphic image, visuospatial skills, motor programming and execution, numerical knowledge, and concentration. The maximum score is 10, and a score of less than 7 indicates cognitive impairment[1].

### Pfeffer Functional Activities Questionnaire (PFAQ)

The Spanish version of the PFAQ includes 11 questions about activities of daily living, with score ranges from 0 to 3 according to the severity of disability in each activity. The maximum score is 33, and a score greater than 6 indicates functional compromise[2].

### Beck Depression Inventory, second version (BDI-II)

The BDI-II is a self-report scale that provides a measure of depression in adolescents over 13 years of age and in adults. It is composed of 21 items including symptoms of sadness, tearfulness, loss of pleasure, feelings of failure and guilt, suicidal thoughts or wishes, pessimism, etc. These symptoms correlate with diagnostic criteria of depressive disorders in the DSM-IV[3] and ICD-10[4]. Scores on the BDI-II range from 0 to 63[5]. The cut-off scores for various degrees of depression are: 0-13, minimal depression; 14-19, mild depression; 20-28, moderate depression; and 29-63, severe depression[6].

### Modified Hachinski index

The Modified Hachinski index was created by Professor Vladimir Hachinski in 1974 to diagnose probable vascular dementia to differentiate it from other types of dementia. It includes questions regarding abrupt or stepwise onset of cognitive impairment, somatic complaints, emotional incontinence, previous history of stroke, current hypertension, focal

neurological symptoms and signs. If less than 4 points are obtained, dementia etiology is likely another type of dementia, such as ADD; a score of 5-6 is mixed pathology dementia, and more than 7 is likely vascular dementia[7]. The index has a sensitivity of 84% and a specificity of 82%[8]. In this study, we used a modified version utilized in other studies in Peru[9].

**Memory Failures of Every Day Life**

The MFE is a 28-item questionnaire that addresses frequency of subjective memory difficulties, such as forgetting things from the day before, forgetting appointments, etc. The categories assessed on the MFE are: 'speaking, reading and writing', 'names and faces', 'actions' and 'learning new things'. Each item is scored on a 0 to 2 point scale ('never or rarely', 'sometimes', 'many times'). Scores below 22 indicate a person with subjective memory complaints.

**References:**


1. Custodio N, García A, Montesinos R, Lira D & Bendezú L. Validación de la prueba de dibujo del reloj-versión de Manos como prueba de cribado para detector demencia en una población adulta mayor de Lima, Perú. Rev Peru Med Exp Salud Publica 2011;28(1):29-34.

2. Quiroga P, Albala C, Klaasen G. [Validation of a screening test for age associated cognitive impairment, in Chile]. *Rev Med Chil* 2004; 132: 467–478.

3. Diagnostic and Statistical Manual of Mental Disorders, Fourth Edition, American Psychiatric Association, 1994.

4. International Statistical Classification of Diseases and Related Health Problems, World Health Organization, 1993.

5. Basic D, Khoo A, Conforti D, Rowland J, Vrantsidis F, Logiudice D, et al. Rowland Universal Dementia Assessment Scale, Mini-Mental State Examination and General Practitioner Assessment of Cognition in a multicultural cohort of community-dwelling older persons with early dementia. Australian Psychologist 2009; 44(1): 40–53. https://doi.org/10.1080/00050060802593593.

6. Rowland JT, Basic D, Storey JE & Conforti DA. (2006). The Rowland Universal Dementia Assessment Scale (RUDAS) and the Folstein MMSE in a multicultural cohort of elderly persons. International Psychogeriatrics 2006; 18(1): 111–120.

https://doi.org/10.1017/S1041610205003133.

7. Storey JE, Rowland JTJ, Conforti DA & Dickson HG. The Rowland Universal Dementia Assessment Scale (RUDAS): A multicultural cognitive assessment scale. International Psychogeriatrics 2004; 16(1): 13–31. https://doi.org/10.1017/S1041610204000043.

8. Nielsen TR, Vogel A, Gade A & Waldemar G. Cognitive testing in non-demented Turkish immigrants - comparison of the RUDAS and the MMSE. Scandinavian Journal of Psychology 2012; 53(6): 455–460. https://doi.org/10.1111/sjop.12018.

9. Custodio N, Herrera-Pérez E, Montesinos R, Lira D & Metcalf T. Brief cognitive tests validated in Peru for detection of cognitive impairment: a systematic mapping of the scientific literature. Dement & Neuropsychol 2020; 14(2): 134-144. doi: 10.1590/1980-57642020dn14-020006.
